# Minimal Sex-Differential Modulation of Reactivity to Pathogens and Toll-Like Receptor Ligands following Infant Bacillus Calmette–Guérin Russia Vaccination

**DOI:** 10.3389/fimmu.2017.01092

**Published:** 2017-09-08

**Authors:** Fatoumatta Darboe, Jane U. Adetifa, John Reynolds, Safayet Hossin, Magdalena Plebanski, Mihai G. Netea, Sarah L. Rowland-Jones, Jayne S. Sutherland, Katie L. Flanagan

**Affiliations:** ^1^Vaccines and Immunity Theme, MRC Unit, Fajara, Gambia; ^2^Biostatistics Consulting Platform, Faculty of Medicine, Nursing and Health Sciences, Monash University, Prahran, VIC, Australia; ^3^Department of Immunology and Pathology, Monash University, Prahran, VIC, Australia; ^4^Monash Institute of Medical Engineering, Monash University, Prahran, VIC, Australia; ^5^Department of Internal Medicine, Radboud Center for Infectious Diseases, Nijmegen University Nijmegen Medical Center, Nijmegen, Netherlands; ^6^Nuffield Department of Medicine, University of Oxford, Oxford, United Kingdom; ^7^School of Medicine, University of Tasmania, Hobart, TAS, Australia

**Keywords:** vaccine, toll-like receptors, non-specific effects, heterologous effects, cytokines, innate immunity, adaptive immunity

## Abstract

Bacillus Calmette–Guérin (BCG), the only licensed vaccine against tuberculosis, has been shown to provide heterologous protection against unrelated pathogens and enhance antibody responses to several routine expanded program on immunization (EPI) vaccines. Understanding these heterologous effects is important for the development of optimal vaccination strategies. We set out to assess the effect of vaccination with BCG Russia of 6-week-old infants on *in vitro* reactivity to a panel of toll-like receptor (TLR) agonists (TLR2, 4, and 7/8) and heat-killed pathogens [*Streptococcus pneumoniae, Candida albicans* (CA), and *Escherichia coli*], and antibody responses to other EPI vaccines compared to BCG naïve infants. We observed no effect of BCG vaccination on innate (TNF-α) or Th2 (IL-4) cytokine responses, but found enhanced CA-specific CD8^+^IFN-γ^+^ responses in BCG vaccinated males and females 1 week after vaccination and decreased IFN-γ:IL4 ratio to SP in females. By 12 weeks (but not 1 week) of post-vaccination, there was significant downmodulation of Th1 cytokine responses in BCG vaccinated infants; and TLR-stimulated IL-10 and IL-17 responses declined in BCG vaccinated females but not males. Significant changes also occurred in the BCG naïve group, mainly at 18 weeks, including decreased Th1 and increased IL-10 responses. The effects at 18 weeks were most likely a result of immune modulation by the intervening EPI vaccines given at 8, 12, and 16 weeks of age. There was no effect of BCG vaccination on EPI antibody levels at either time point. Taken together, our results support minimal early heterologous immune modulation by BCG Russia vaccination that did not persist 12 weeks after vaccination.

## Introduction

Tuberculosis (TB) is a significant public health problem with approximately two billion people infected globally (1). Bacillus Calmette–Guérin (BCG) is the only licensed vaccine against TB, and while it provides good protection against disseminated TB in childhood, it has poor efficacy against adult pulmonary TB (2, 3). Several reasons have been given for the variability in efficacy throughout the world, including, but not limited to, different strains of BCG vaccine (4–6), geographical location (7), and age at vaccination (8, 9).

Neonates have Th2-biased immunity and are thus susceptible to microbial infections requiring Th1 immunity for clearance, such as TB (10–12). Despite this, BCG vaccination at birth can induce a robust adaptive immune response, inducing polyfunctional Th1 cells (13–15) and Th17 cells (8, 16–18). Besides inducing a classical antigen-specific T cell response, BCG vaccination also induces an innate immune response involving dendritic cell activation, natural killer (NK) and NK T cells, and neutrophils (19–22). The innate immune system plays a critical protective role against infectious diseases throughout life, inducing a rapid immunological response against invading pathogens prior to the generation of adaptive immunity. This is particularly important in early life when adaptive immunity is poorly developed.

Toll-like receptors (TLRs) expressed by host immune cells recognize pathogen-associated molecular patterns (PAMPs), required for microbial recognition by macrophages and dendritic cells (23, 24). TLR ligands have been used in murine models as vaccine adjuvants, leading to enhanced Th1 responses (25). Certain TLR polymorphisms have been associated with an increase in BCG induced Th-1 type responses in whole blood 10 weeks after BCG vaccination in South African infants (26). Furthermore, BCG has been shown to induce epigenetic reprogramming of innate immune cells toward a pro-inflammatory profile characterized by increased IFN-γ, TNF-α, and IL-1β in both murine models and adult European individuals (27). These studies showed that innate cells exhibit characteristics of adaptive immunity or adaptation to secondary infection in a process termed “trained immunity.” This immune training by BCG vaccination was further able to enhance innate immunity to other unrelated pathogens including *Candida albicans* (CA), *Staphylococcus aureus*, and *Streptococcus pneumoniae* (SP) (27).

Epidemiological studies dating back to the 1940s have suggested that BCG vaccination provides protection against unrelated infections, and vaccination is associated with a decrease in all-cause mortality in certain settings (28). A meta-analysis of clinical trial data from the US and UK in the 1940s and 1950s estimated a 25% reduction from diseases other than TB when BCG was given (29). Randomized controlled trials in Guinea Bissau have shown that BCG vaccination of low birth weight neonates is associated with an almost 50% reduction in all-cause mortality in the first few weeks of life, mainly from unrelated infections (30). In an analysis of almost half a million childhood hospitalization episodes in the official Spanish registry, BCG vaccination was associated with a 32.4–66.6% reduction in hospitalization due to respiratory infection, and a 52% reduction in sepsis in <1-year olds (31). Similarly, data from two large cohorts of children (*n* = 58,021 and 93,301) from 19 countries found a 17–37% reduction in acute lower respiratory tract infection in BCG vaccinated children (32). Other evidence for non-specific effects of BCG include its widespread use as a therapeutic agent against bladder cancer (33) and as an adjunctive therapy for malignant melanoma (34). BCG has also been shown to boost antibody responses to other expanded program on immunization (EPI) vaccines supporting an adjuvant effect in humans (15, 35).

Interestingly, non-specific effects of vaccines have been shown to manifest differently in the sexes, with females generally being more susceptible than males to the non-specific effects of vaccination (36–38). Indeed, the protection against death in the low birth weight studies occurred in the first 3 days of life in males and after 1 week in females, suggesting different mechanisms (30). Despite these important findings, the underlying immunological mechanisms are still not understood.

Herein, we conducted a randomized trial aimed at assessing the effect of BCG vaccination of 6-week-old Gambian infants on *in vitro* responses to toll-like receptor (TLR) ligands, unrelated pathogens, and antibody responses to EPI vaccines compared to a BCG naïve control group. Our results support early enhanced CA-specific IFN-γ responses in BCG vaccinated infants but no effect on TNF-α or EPI vaccine antibody responses. Our findings contribute to the relatively scarce evidence for heterologous immunological effects of BCG vaccination in humans.

## Materials and Methods

### Study Site and Subject Recruitment

Expectant mothers, presenting at the Sukuta Health Centre (a peri-urban setting in The Gambia) for antenatal care were sensitized about the study. Their children were recruited into the study upon birth or when they presented for BCG vaccination (within 48 h of birth). BCG vaccination was withheld for those that agreed to participate (Table 1). Exclusion criteria included low birth weight (<2.5 kg), congenital defects, and multiple births.

**Table 1:** Vaccines given throughout the study.

	BCG group	Control group
Birth	OPV, HepB	OPV, HepB
6 weeks	**BCG**	Nil
8 weeks	Penta 1 (Hib, HepB, DTwP), PCV-13, OPV	Penta 1 (Hib, HepB, DTwP), PCV-13, OPV
12 weeks	Penta 2 (Hib, HepB, DTwP), PCV-13, OPV	Penta 2 (Hib, HepB, DTwP), PCV-13, OPV
16 weeks	Penta 3 (Hib, HepB, DTwP), PCV-13, OPV	Penta 3 (Hib, HepB, DTwP), PCV-13, OPV
18 weeks	TST	TST then **BCG**

*All vaccines given to study infants from birth to the end of the study at 18 weeks of age*.

*OPV, oral polio vaccine; HBV, hepatitis B vaccine; BCG, bacillus Calmette–Guérin vaccine; Penta, pentavalent vaccine; PCV-13, 13-valent pneumococcal conjugate vaccine; DTwP, diphtheria-tetanus-whole cell pertussis vaccine; Hib, Haemophilus influenzae group b vaccine; TST, tuberculin skin test*.

### Ethics Statement

The Scientific Coordinating Committee (SCC) at the Medical Research Council (MRC) Unit, The Gambia and the MRC-Gambian Government Joint Ethics Committee approved this study (study number SCC 1233). Written informed consent was provided by the parent/guardian of participating infants in accordance with the Declaration of Helsinki.

### Blood Sampling and Vaccination

**Figure 1:**
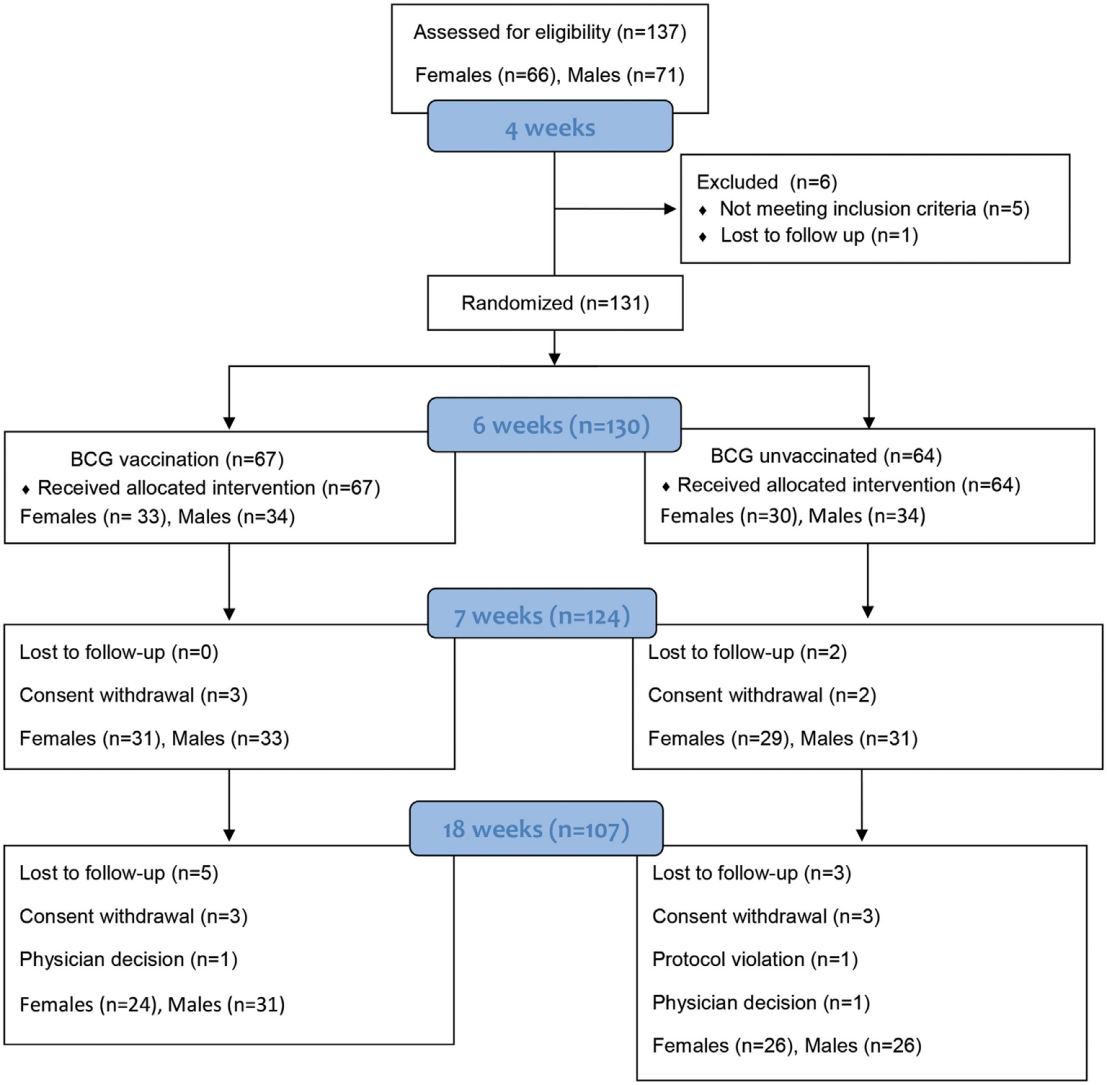
Participant numbers and drop outs. Numbers of study infants throughout the study from recruitment at birth, to enrollment and randomization, and follow-up until 18 weeks of age. Infants were bled at three time points—Visit 1 (V1) at 6 weeks of age (baseline), visit 2 (V2) at 7 weeks (+1 week) and visit 3 (V3) at 18 weeks of age (+12 weeks).

Infants were randomized at 6 weeks of age into one of two groups: 0.1 mL intradermal vaccination with BCG vaccine (Russian strain, SSI India) (BCG group) or no vaccination (Control group) to be given at 6 weeks of age. All infants received hepatitis B vaccine (HepB) (Serum Institute of India) and oral polio vaccine (OPV) (Sanofi Pasteur, France) at birth in accordance with the EPI schedule of The Gambia. At 8, 12, and 16 weeks, all infants received the pentavalent [diphtheria–tetanus–whole cell pertussis, *Haemophilus influenzae B* (Hib), HepB] vaccine (Panacea biotech, India), OPV, and the 13-valent pneumococcal conjugate vaccine (PCV-13) (Table 1). At 18 weeks of age, all infants were tested by standard tuberculin skin test (TST) for mycobacterial reactivity by injecting 0.1 mL containing 2 U of tuberculin (Serum Staten Institut, Denmark) intradermally. Induration was read longitudinally and transversely by a trained nurse at 48–72 h to determine the average induration. The infants in the BCG naïve control group were then vaccinated with BCG at 18 weeks of age. A maximum of 5 mL of venous whole blood was collected into EDTA tubes at 6 weeks (prior to BCG vaccination of group 1) [Visit 1 (V1)], 7 weeks (1 week post-BCG vaccination) [Visit 2 (V2)], and 18 weeks (12 weeks post-BCG vaccination) [Visit 3 (V3)] (Figure 1).

### Peripheral Blood Mononuclear Cell (PBMC) Separation and Cryopreservation

EDTA blood was spun at 1,500 rpm for 10 min and 1 mL of plasma collected and stored at −20°C until use. The remaining whole blood was diluted 1:1 with RPMI (Sigma, UK) and PBMC separation was performed *via* density gradient centrifugation on a Lymphoprep (Fresenius Kabi Norge AS, Norway) cushion. Harvested PBMCs were washed twice in RPMI and cells counted using Trypan blue exclusion. For every 10 million live cells, 500 µL of fetal calf serum (FCS) (Gibco, Life technologies, USA) was added, cells were left on ice for 30 min and 500 μL of 20% dimethyl sulfoxide (Sigma-Aldrich, UK) in FCS was added before cells were transferred into a Nalgene^®^ Mr Frosty container (Sigma-Aldrich) and stored at −70°C then transferred to liquid nitrogen within 24 h.

### PBMC Thawing

Peripheral Blood Mononuclear Cells were thawed rapidly at 37°C in a water bath. Cells were then resuspended slowly in warm 10% FCS in RPMI (R10) prior to washing twice. Viability counts were done on the samples before being resuspended in R10 and 25 U/mL of benzonuclease (Sigma, UK). PBMCs were then rested at 37°C 5% CO_2_ for 6 h.

### *In Vitro* Culture Conditions

0.1 × 10^6^ PBMCs in 100 µL of culture medium (2% penicillin/streptomycin, 1% l-glutamine, and 10% FCS in RPMI) were stimulated in 96-well round bottom plates for 16 h with the following antigens: purified protein derivative (PPD) (10 µg/mL, Serum Staten Institute, Denmark), heat-killed *Listeria monocytogenes* (HKLM) (TLR2 agonist; 10^9^/mL, Invivogen), lipopolysaccharide (LPS) (TLR4 ligand; 1 µg/mL, Invivogen), CLO-75 (TLR7/8 agonist; 10 µg/mL, Invivogen); and heat-killed *Streptococcus pneumonia* (SP) (10^5^ cells/mL), CA (10^5^ cells/mL), or *Escherichia coli* (EC) (10^6^ cells/mL). PMA/ionomycin (0.1 μg/1 μg/mL, Sigma, UK) was used as a positive control, and RPMI as a negative control. Supernatants were collected after the addition of 100 µL of RPMI and centrifugation at 1,500 rpm for 10 min. Supernatants were stored at −20°C until needed for cytokine analysis.

### Multiplex Cytokine Assays

Cytokines in culture supernatants were assessed using Bioplex Pro custom made kits according to the manufacturer’s instructions using a 1:2 dilution of reagents (Bio-Rad, Belgium). The cytokines analyzed were IFN-γ, TNF-α, IL-2, IL-4, IL-10, IL-17, and IL-12(p70). Defrosted culture supernatants were centrifuged for 5 min to pellet any precipitate and left on ice until use. Coupled magnetic beads were diluted 1:2 with assay buffer, and added to pre-wet 96-well filter plates. Plates were washed using a vacuum manifold station. 50 µL of samples and standard were added and the plate incubated on a shaker at 300 rpm for 30 min at room temperature. Detection antibodies were diluted 1:2 with detection antibody diluents, 25 µL added to the plate after three washes, and incubated for 30 min on a shaker at room temperature. The plate was washed three times using the vacuum manifold before addition of 50 µL of streptavidin, and then incubated for 10 min on a shaker. 125 µL of assay buffer was added and plates left on a shaker until ready to be read using the Bioplex 200 system, Luminex x-map technology, and Bioplex pro software version 4.0 (Bio-Rad, Belgium).

All values less than the lowest value of the standard within the standard curve were given the value of the lowest standard and all values higher than the highest standard were given the value of the highest standard. The range in picograms per milliliter for each analyte is as follows: IL-2 (0.88–14,426), IL-4 (0.35–5,693), IL-10 (1.45–23,718), IL-12p70 (2.65–43,449), IL-17 (2.33–38,209), IFN-γ (2.05–33,560), and TNF-α (6.69–109,604). Background (unstimulated) values were subtracted from antigen-stimulated values to determine the antigen-specific response.

### Intracellular Cytokine Staining

0.5 × 10^6^ PBMCs were stimulated with RPMI, PMA/ionomycin, PPD, a peptide pool of the TB-specific antigens ESAT-6/CFP-10 (2.5 µg/mL, ProImmune, UK), heat-killed SP (10^5^ cells/mL), *C. albicans* (10^5^ cells/mL), or *E. coli* (10^6^ cells/mL) per test. Cells were incubated at 37°C 5% CO_2_ for 18 h with Brefeldin A (10 µg/mL) added after the first 2 h of incubation. Cells were stained with a live/dead aqua yellow stain (Invitrogen, USA), incubated for 10 min at room temperature before staining with a surface marker cocktail consisting of either CD3-PerCP Cy5.5 or CD3 APC efluor 750 and CD8 APC efluor 780. Cells were incubated for 30 min at room temperature and washed with 1 mL of PBS/1% FCS/0.2% Sodium Azide (FACS buffer). Supernatant was removed and cells washed in perm/wash solution (Ebioscience, UK). Permeabilization was then performed using fix/perm solution (Ebioscience) and cells incubated for 20 min at 4°C before centrifugation at 1,800 rpm for 5 min. A cocktail of intracellular cytokines IL-2 FITC, IL-17 PE, TNF-α PE-Cy7, IL-10 efluor-450, and IFN-γ APC (all from ebioscience) was added, and cells incubated for 30 min at room temperature. Cells were washed with perm/wash solution and 300 µL FACS buffer was added before acquisition. At least 100,000 lymphocytes were acquired for each sample on a CyanADP (Beckman Coulter, USA) flow cytometer using Summit v4 software and analyzed using FlowJo v10.0.2 (Tree Star, USA) according to the gating strategy shown (Figures S1 and S2 in Supplementary Material).

### Vaccine Antibody Assays

Stored plasma samples were used to measure antibody levels to polio 1 and 3 using the antibody neutralizing assay as described previously (39). Briefly, Hep-2 Cincinnati cells were cultured with different dilutions of the virus and subject plasma, cytopathic effect of the virus on the plasma was assessed after 5 days incubation. Hepatitis B antibody levels were measured using a commercial ELISA kit (Diasorin, Italy) as per the manufacturer’s instructions. Diphtheria, tetanus, and pertussis antibodies were assessed in house using a multiplex immunoassay developed by the RIVM in the Netherlands as described previously (40), and antibody levels were measured using the Bioplex 200 system (Bio-Rad, Belgium).

### Statistical Analysis

Cytokine multiplex, intracellular staining (ICS), and antibody data were analyzed by fitting a linear mixed model using restricted maximum likelihood. Bleed, vaccine group and sex, and their interactions were fitted as fixed effects, and infant and sample within infant, were fitted as random effects. The *F*-test was used to test for interactions between the fixed effects, in particular the two-way group-by-bleed and three-way sex-by-group-by-bleed interactions. Comparisons between treatment groups, and sexes within treatment groups, at each time point, were based on *t*-tests utilizing the predicted means and SEs of differences recovered from the fitted mixed model. Comparisons were conducted at the 5% significance level with no adjustments for multiplicity of outcome variables or comparisons. Ratios of cytokines were intrinsically skewed and required logarithmic transformation prior to analysis, whereas diagnostic plots of residuals indicated that the cytokine data did not require log transformation. Logarithmic transformations were also required to overcome heterogeneity of variance in the antibody data. Data were analyzed using GenStat 17 (VSN International), R 3.1.2 (www.r-project.org), and Stata version 12.1 (StataCorp LP, USA).

## Results

### Infant Characteristics

137 infants were recruited into the study at birth with males and females randomized separately (Figure 1). The groups were of equivalent weight-for-age *z* scores at recruitment (data not shown). At 6 weeks (range 5–7 weeks), 131 babies remained in the study and were randomized to one of the two study groups: 67 infants (34 males and 33 females) received BCG and 64 (35 males and 29 females) were randomized to the control group and received BCG at 18 weeks upon study completion (Figure 1).

### Lack of Enhanced *In Vitro* PPD Reactivity, but Greater TST Responses, in BCG Vaccinated Infants

Tuberculin skin test reactivity and *in vitro* PPD assays were used to assess mycobacterial responses post-vaccination. The BCG vaccinated group had greater TST reactivity than the naïve control group (Table 2). Thus, while only 7 of 52 (13.5%) BCG naïve infants had a positive TST response (any induration > 1 mm), 32 of 53 (60.4%) BCG vaccinated infants were TST positive. The median induration in the unvaccinated infants was 0 (IQ range 0, 0) further indicating low level TST activity in this group, as compared to the BCG vaccinated group who had a median induration of 4.5 mm (IQ range 0, 10.5) (Control group vs BCG group *p* < 0.0001). The BCG vaccinated males had greater induration than females (M 8.5 mm, F 1.25 mm, M vs F *p* = 0.014) and responded more frequently than females (M = 69%, F = 50%).

**Table 2:** Tuberculin skin test reactivity at 18 weeks of age.

	Number tested	Number responders (%)	Median induration in mm (IQ range)
All naïve controls	52	7 (13.5)	0 (0, 0)
Naïve control females	26	4 (15.4)	0 (0, 0)
Naïve control males	26	3 (11.5)	0 (0, 0)
All bacillus Calmette–Guérin (BCG) vaccinated	53	32 (60.4)	4.5 (0, 10.5)
BCG vaccinated females	24	12 (50)	1.25 (0, 5)
BCG vaccinated males	29	20 (69)	8.5 (0, 12.5)

**Figure 2:**
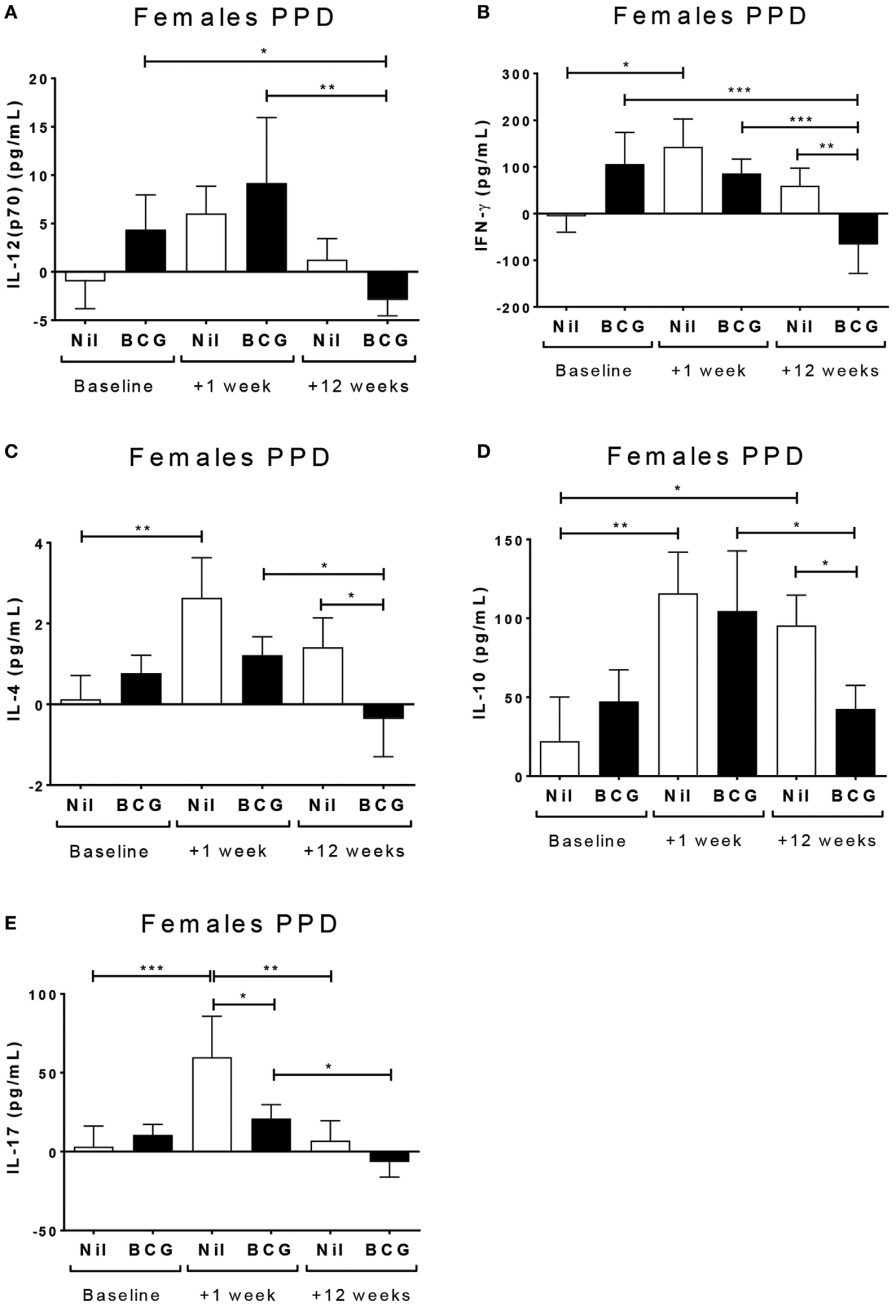
Sex-differential changes in purified protein derivative (PPD) reactivity. Figure shows comparisons of means within sexes for analyses with significant effects (main effect of interactions) involving bleed for the cytokine responses to stimulation with PPD. Significant effects were only observed in females and not male infants. The bacillus Calmette–Guérin (BCG) vaccinated females had detectable responses at baseline, in particular IFN-γ and IL-10, but these were not boosted further 1 (V2) or 12 weeks (V3) post-vaccination. Indeed, by V3, there was a significant decline in IL-12(p70) **(A)**, IFN-γ **(B)**, IL-4 **(C)**, IL-10 **(D)**, and IL-17 **(E)** at V3 in the BCG vaccinated females. Mean baseline levels were low at baseline for the BCG naïve group, but increased significantly for IFN-γ **(B)**, IL-4 **(C)**, and IL-17 **(E)** by V2 (+1 week). A fitted linear mixed model of cytokine values was used to determine significant differences by vaccine group, bleed, and sex. *p < 0.05, **p < 0.01, and ***p < 0.001. The figures show the arithmetic mean and SEM for cytokines in picograms per milliliter. Data shown for *n* = 93 infants (26 BCG vaccinated females, 18 BCG vaccinated males, 24 control females, and 25 control males).

Surprisingly, there was no evidence of a boosting of PPD reactivity in the BCG vaccinated group at V2 or V3 for any of the cytokines tested in culture supernatants (Table S1 in Supplementary Material). On the contrary, in BCG vaccinated females but not males the PPD-specific IL-12(p70) (*p* = 0.0025) (Figure 2A), IFN-γ (*p* = 0.0003) (Figure 2B), IL-4 (*p* = 0.0018) (Figure 2C), IL-10 (*p* = 0.004) (Figure 2D), and IL-17 (*p* = 0.0003) (Figure 2E) had all declined by V3 compared to V2. By contrast, the BCG naïve group had an increase in IFN-γ (*p* = 0.0164) (Figure 2A), IL-4 (*p* = 0.0018) (Figure 2C), IL-10 (*p* = 0.004) (Figure 2D), and IL-17 (*p* = 0.0003) (Figure 2E) at V2 compared to baseline.

Intracellular staining by flow cytometry was performed to characterize CD4 and CD8 T cells, and innate cells (CD3^−^ cells) expressing IL-2, IL-17, TNF-α, IL-10, IFN-γ, or combinations of these cytokines intracellularly (Figures S1 and S2 in Supplementary Material). This failed to show any significant sex, group, or time interactions for PPD-specific CD4^+^, CD8^+^, or CD3^−^ ICS responses for any of the cytokines in BCG vaccinated or control infants (data not shown).

### No Effects of BCG Vaccination on Innate Cytokine Responses

Levels of the innate pro-inflammatory cytokine TNF-α were analyzed in the TLR ligand and pathogen culture supernatants after overnight stimulation. While robust TNF-α responses were detected to the TLR ligands (Table S2 in Supplementary Material) and heat-killed pathogens (Table S3 in Supplementary Material), there was no difference in TNF-α responses between BCG vaccinated and naïve infants nor between males and females. In keeping with the soluble cytokine data, there was no effect of vaccine group, bleeding time point or sex on TNF-α responses by ICS of CD4^+^, CD8^+^, or CD3^−^ cells (data not shown).

### Sex-Differential Effects of BCG Vaccination on Th1/Th2 Polarizing Cytokines

**Figure 3:**
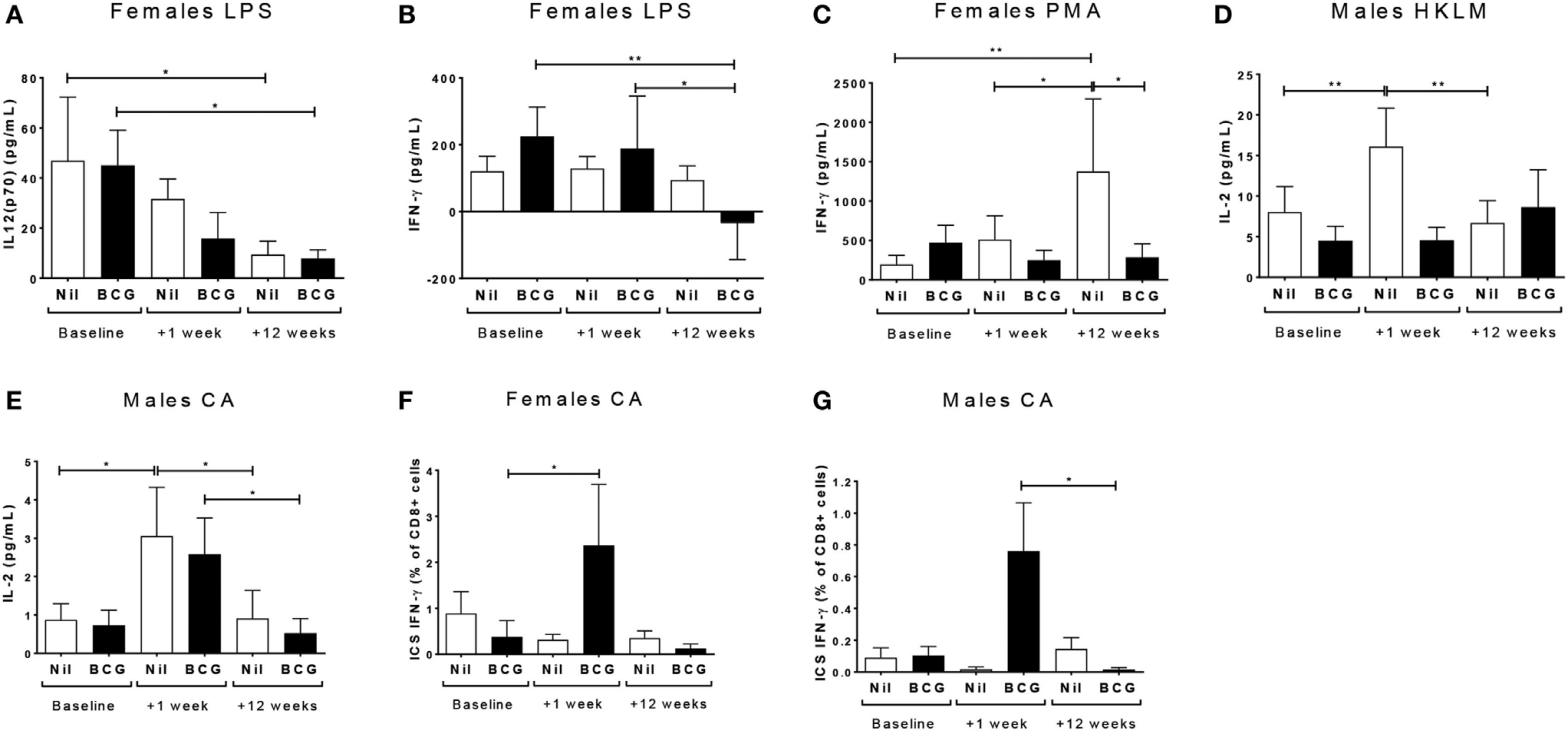
Th1 polarizing cytokine responses. The Th1 polarizing cytokines IL-12(p70), IL-2, and IFN-γ were measured in culture supernatants and IFN-γ was measured in CD4, CD8 T cells, and non-T cells by flow cytometry. Only significant effects by vaccine group, bleed, and sex using a fitted linear mixed model of cytokine values are shown. These indicated effects on IL-12(p70) **(A)** and IFN-γ **(B)** responses to LPS in females; IFN-γ responses to PMA in females **(C)**; IL-2 responses to HKLM **(D)** and CA **(E)** in males; and intracellular staining (ICS) IFN-γ CD8^+^ T cell responses in to CA females **(F)** and males **(G)**. *p < 0.05 and **p < 0.01. The figures show the arithmetic mean and SEM for cytokines in picograms per milliliter **(A–E)** or % positive cells of the gated CD8^+^ population **(F,G)**. CA, *Candida albicans*; HKLM, heat-killed *Listeria monocytogenes*; LPS, lipopolysaccharide; PMA, phorbol myristate acetate/ionomycin. Data shown for *n* = 79 infants [23 bacillus Calmette–Guérin (BCG) vaccinated females, 15 BCG vaccinated males, 21 control females and 20 control males].

IL-12(p70) (V1 vs V3 *p* = 0.0355) and IFN-γ (V1 vs V3 *p* = 0.0018; V2 vs V3 *p* = 0.0232) responses declined to the TLR4 ligand LPS in BCG vaccinated females, and IL-12(p70) (V1 vs V3 *p* = 0.0129) but not IFN-γ declined in the BCG naïve control females too (Figures 3A,B). BCG naïve females showed an increase in IFN-γ production to PMA/ionomycin by V3 (V1 to V3: *p* = 0.0019; and V2 to V3: *p* = 0.0334), while the BCG vaccinated females did not (Figure 3C). BCG naïve, but not vaccinated, males had increased IL-2 production to both the TLR2 ligand HKLM (V1 vs V2 *p* = 0.0057) (Figure 3D) and CA (V1 vs V2 *p* = 0.0207) (Figure 3E) at V2, which declined by V3 (HKLM V2 vs V3 *p* = 0.0005; CA V2 vs V3 *p* = 0.026). The BCG vaccinated males also had an increase in IL-2 to CA at V2, which did not quite reach significance (*p* = 0.0512), but declined significantly by V3 (V2 vs V3 *p* = 0.0498) (Figure 3E). The only effect on intracellular cytokine production by flow cytometry after stimulating with the TLR ligands and heat-killed pathogens was an increase in IFN-γ from CD8^+^ T cells after CA stimulation in the BCG vaccinated females at V2 compared to V1 (*p* = 0.0316) (Figure 3F). The similar increase in CA-specific CD8^+^IFN-γ^+^ responses from V1 to V2 was not significant in the males, but the level at V2 was significantly higher than at V3 (*p* = 0.0151) (Figure 3G).

**Figure 4:**
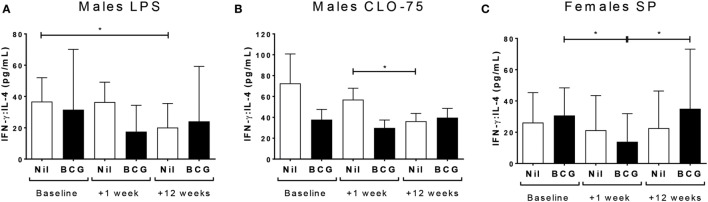
Th1:Th2 cytokine balance. IFN-γ:IL-4 in LPS **(A)** and CLO-75 **(B)** culture supernatants declined by V3 (+12 weeks) in bacillus Calmette–Guérin (BCG) naïve, but not vaccinated, males. IFN-γ:IL-4 to SP declined at V2 (+1 week) in BCG vaccinated, but not naïve, females, then increased to baseline levels by V3 (+12 weeks) **(C)**. *p < 0.05. The figures show the geometric mean and 95% CI for cytokines in picograms per milliliter. LPS, lipopolysaccharide; SP, *Streptococcus pneumoniae*. Data shown for *n* = 79 infants (23 BCG vaccinated females, 15 BCG vaccinated males, 21 control females, and 20 control males).

While the Th2 cytokine IL-4 was not affected by vaccine group or sex in culture supernatants, the ratio of IFN-γ to IL-4, indicative of the Th1:Th2 cytokine balance, was affected. Thus, BCG naïve males had decreased IFN-γ:IL-4 at V3 to TLR4 (LPS) (V1 vs V3 *p* = 0.039) (Figure 4A) and TLR7/8 (CLO-75) stimulation (V2 vs V3 *p* = 0.0194) (Figure 4B); and BCG vaccinated females had decreased IFN-γ:IL-4 to SP at V2 compared to baseline (V1 vs V2 *p* = 0.0128) which increased again by V3 (V2 vs V3 *p* = 0.0227) (Figure 4C; Table 3; Tables S2 and S3 in Supplementary Material).

**Table 3:** Summary of all significant sex-by-bleed-by-group interactions for the whole blood culture and intracellular staining assays.

	Innate cytokines	TH1 cytokines	TH2 cytokines	TH1:TH2 ratio	Anti-inflammatory	Pro- to anti-inflammatory	TH17 cytokines

	TNF-α	IL-12(p70), IL-2, IFN-γ	IL-4	IFN-γ:IL-4	IL-10	IFN-γ:IL-10, TNF:IL-10	IL-17
**Visit 2 (+1 week)**							
Naïve females	No effect	No effect	No effect	No effect	↑ to CA	↓ to SP	No effect
Bacillus Calmette–Guérin (BCG) females	No effect	↑ CD8^+^IFN-γ^+^ to CA	No effect	↓ to SP	No effect	No effect	No effect
Naïve males	No effect	↑ to TLR2 and CA	No effect	No effect	No effect	No effect	↑ to TLR2
BCG males	No effect	↑ CD8^+^IFN-γ^+^ to CA	No effect	No effect	No effect	No effect	No effect
**Visit 3 (+12 weeks)**							
Naïve females	No effect	↓ to TLR4, ↑ to PMA	No effect	No effect	↑ to CA	↓ to SP, EC	No effect
BCG females	No effect	↓ to TLR4	No effect	↑ to SP	↓ to TLR4	No effect	↓ to TLR2
Naïve males	No effect	↓ to TLR2 and CA	No effect	↓ to TLR4 and TLR7/8	No effect	No effect	No effect
BCG males	No effect	↓ to CA	No effect	No effect	No effect	↓ to SP, TLR2, TLR4	No effect

*Table summarizing all significant interactions in the linear mixed model analysis showing that cytokine changes are sex, group, and time differential*.

### Sex-Differential Effects on the Anti-inflammatory Cytokine IL-10

**Figure 5:**
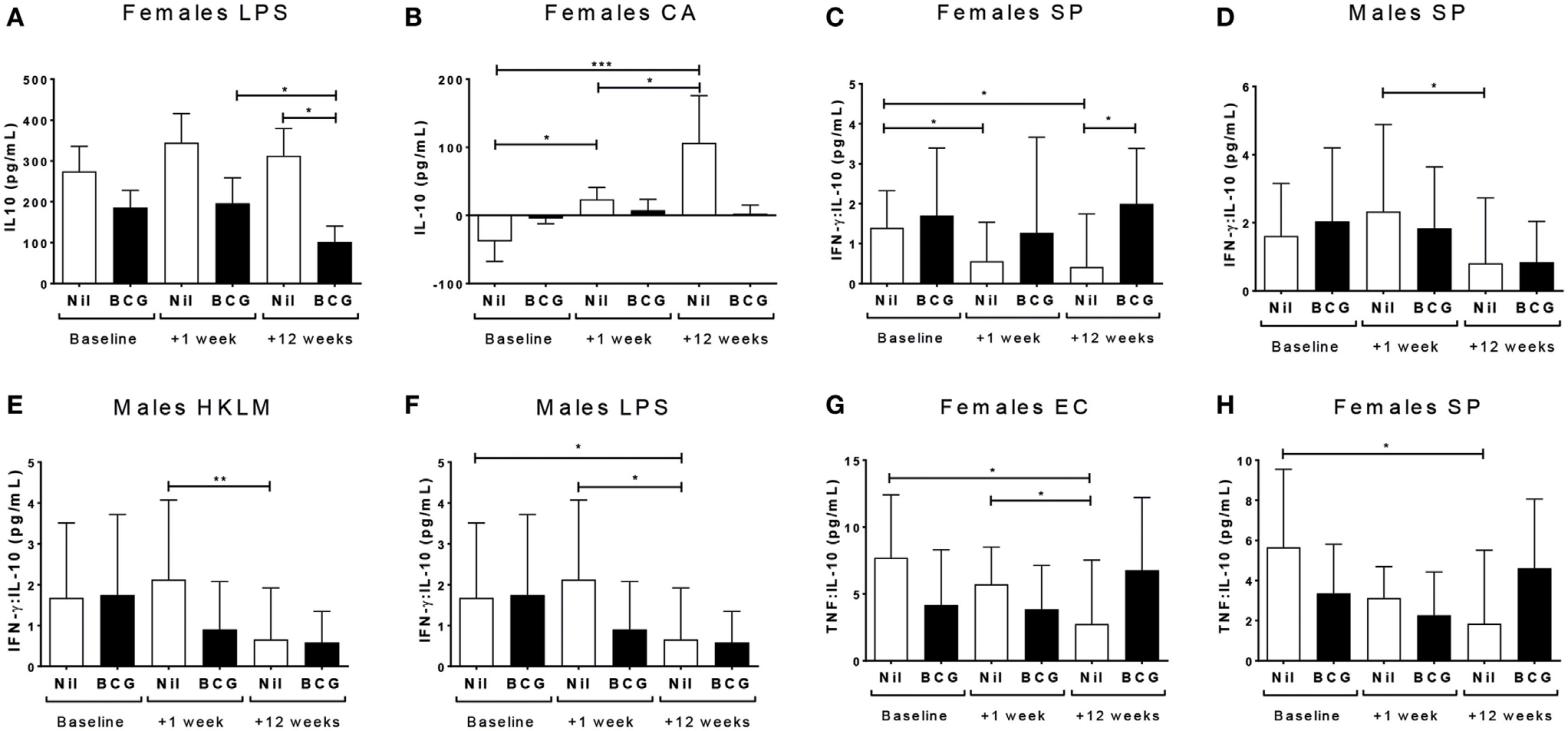
Effects on the anti-inflammatory cytokine IL-10. The modeling analysis showed differences by vaccine group, bleed, and sex for female IL-10 responses to LPS **(A)** and CA **(B)**. IFN-γ:IL-10 ratios declined in SP cultures in bacillus Calmette–Guérin (BCG) naïve females **(C)**; and declined in SP **(D)**, HKLM **(E)**, and LPS **(F)** cultures in BCG naïve males. TNF-α:IL-10 levels decreased by V3 (+12 weeks) in EC **(G)** and SP **(H)** cultures in BCG naïve females. *p < 0.05, **p < 0.01, and ***p < 0.001. The figures show the arithmetic mean and SEM for individual cytokines in picograms per milliliter, and geometric mean and 95% CI for log transformed cytokine ratios. CA, *Candida albicans*; EC, *Escherichia coli*; HKLM, heat-killed *Listeria monocytogenes*; LPS, lipopolysaccharide; SP, *Streptococcus pneumoniae*. Data shown for *n* = 79 infants (23 BCG vaccinated females, 15 BCG vaccinated males, 21 control females, and 20 control males).

IL-10 is an anti-inflammatory cytokine that can be produced by T helper 1 (Th1), Th2, and regulatory T cells (41). IL-10 responses to TLR4 (LPS) declined in the BCG vaccinated females by V3 (V2 vs V3 *p* = 0.0482) (Figure 5A), while IL-10 responses to CA increased in the BCG naïve but not vaccinated females at V2 with a further increase at V3 (V1 vs V3 *p* = 0.0001, V2 vs V3 *p* = 0.0217) (Figure 5B; Tables S2 and S3 in Supplementary Material). There was no effect of vaccine group, bleeding time point, or sex on ICS IL-10 responses to any antigen (data not shown).

Bacillus Calmette–Guérin naïve females had a decrease in IFN-γ:IL-10 to SP at V2 and V3 (V1 vs V2 *p* = 0.0191; V1 vs V3 *p* = 0.0141) (Figure 5C), and BCG naïve males likewise had a decline in IFN-γ:IL-10 to SP at V3 (V2 vs V3 *p* = 0.0472) (Figure 5D). BCG naïve males also had a decline in IFN-γ:IL-10 to TLR2 (HKLM) (V2 vs V3 *p* = 0.017) (Figure 5E) and TLR4 (LPS) *in vitro* at V3 (V1 vs V3 *p* = 0.0342; V2 vs V3 *p* = 0.0163) (Figure 5F). Furthermore, the ratio of TNF-α to IL-10 in EC (V1 vs V3 *p* = 0.005; V2 vs V3 *p* = 0.0457) (Figure 5G) and SP cultures (V1 vs V3 *p* = 0.0028) (Figure 5H) decreased by V3 in the naïve females, but remained constant in those who received BCG. This effect was not observed in male infants (Table 3).

### Sex-Differential Effects of BCG Vaccination on IL-17 Responses to TLR2 Ligand Stimulation

**Figure 6:**
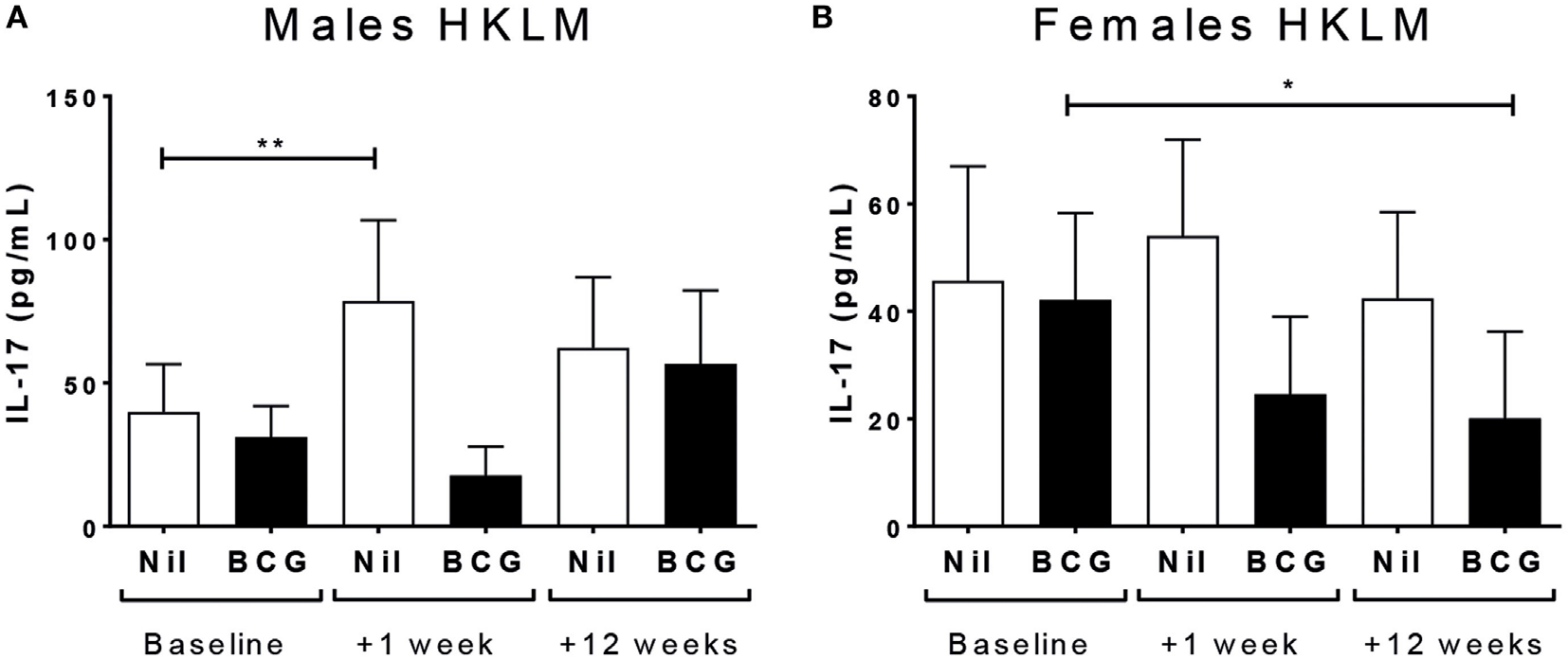
IL-17 responses. IL-17 responses in heat-killed *Listeria monocytogenes* (HKLM) cultures increased in bacillus Calmette–Guérin (BCG) naïve males at V2 (+1 week) **(A)**, and decreased in HKLM cultures in BCG vaccinated females **(B)**. *p < 0.05 and **p < 0.01. The figures show the arithmetic mean and SEM for IL-17 in picograms per milliliter. Data shown for *n* = 79 infants (23 BCG vaccinated females, 15 BCG vaccinated males, 21 control females, and 20 control males).

IL-17 production following TLR2 ligand (HKLM) stimulation increased at V2 in the BCG naïve (V1 vs V2 *p* = 0.0407), but not vaccinated, males (Figure 6A); and HKLM-specific IL-17 declined at V3 in BCG vaccinated (V1 vs V3 *p* = 0.0165), but not naïve, females (Figure 6B). There was no effect of vaccine group or sex on IL-17 responses for any of the other stimuli (Tables S2 and S3 in Supplementary Material). IL-17 reactivity in CD4^+^, CD8^+^, or CD3^−^ cells by ICS was not affected by BCG vaccination or sex at any time point (Table 3).

### No Effect of BCG Vaccination at 6 Weeks on Antibody Responses to Other EPI Vaccines

Since BCG has been shown to boost antibody responses to other EPI vaccines, even when given before the vaccine in question (35), we measured antibody levels to hepatitis B vaccine, poliovirus serotypes 1 and 3, diphtheria toxoid, tetanus toxoid (TT), and pertussis toxoid. There was significant induction of antibody responses to all vaccines by V3, but not V2, compared to V1 (Table 4). All infants except for one had protective antibody levels to TT at V1, confirming a benefit of maternal vaccination, as expectant mothers in The Gambia receive two doses of TT vaccine during the second and third trimesters. No additional boosting effect was observed in the BCG vaccinated group compared to the naïve group for any of the vaccines. The only sex difference was higher HepB IgG levels in control males compared to control females at V3 (*p* = 0.040), while no such difference was observed in the BCG vaccinated group.

**Table 4:** Antibody titers to EPI vaccines in the bacillus Calmette–Guérin (BCG) vaccinated and naïve infants.

Vaccine type (unit)	Visit	BCG vaccinated group					BCG naïve control group				
		Males	Boost from baseline	Females	Boost from baseline	Males vs females	Males	Boost from baseline	Females	Boost from baseline	Males vs Females
	
		Median (IQR)	*p*-Value	Median (IQR)	*p*-Value	*p*-Value	Median (IQR)	*p*-Value	Median (IQR)	*p*-Value	*p*-Value
Polio 1 (titers)	1	24 (8, 160)	32 (8, 112)	NS	16 (8, 112)	16 (8, 48)	NS
	2	48 (16, 256)	NS	48 (20, 512)	NS	NS	24 (16, 256)	NS	32 (16, 64)	NS	NS
	3	256 (32, 1,024)	0.0003	768 (128, 1,024)	<0.0001	NS	256 (160, 512)	<0.0001	192 (64, 1,024)	<0.0001	NS

Polio 3 (titers)	1	8 (8, 80)	8 (8, 14)	NS	(8, 8)	8 (8, 12)	NS
	2	24 (8, 128)	NS	8 (8, 32)	NS	NS	8 (8, 40)	NS	8 (8, 16)	NS	NS
	3	256 (128, 512)	256 (56, 640)	<0.0001	NS	256 (32, 1,024)	<0.0001	256 (10, 512)	<0.0001	NS

HepB (IU/mL)	1	10 (10, 10)	10 (10, 10)	NS	10 (10, 10)	10 (10, 10)	NS
	2	10 (10, 10)	NS	10 (10, 10)	NS	NS	10 (10, 10)	NS	10 (10, 10)	NS	NS
	3	483 (364, 1,000)	<0.0001	650 (394, 1,000)	<0.0001	NS	1,000 (393, 1,000)	353 (241, 1,000)	<0.0001	0.04

Diphtheria toxoid (IU/mL)	1	0.01 (0.01, 0.02)	0.01 (0.01, 0.02)	NS	0.01 (0.00, 0.03)	0.01 (0.00, 0.01)	NS
	3	2.6 (1.4, 3.7)	<0.0001	2.7 (1.9, 4.1)	<0.0001	NS	2.8 (1.7, 4.4)	<0.0001	1.8 (1.2, 3.0)	<0.0001	NS

Pertussis toxoid (EU/mL)	1	3.0 (1.5, 9.6)	5.0 (1.2, 9.5)	NS	4.6 (1.8, 8.8)	4.1 (1.6, 9.7)	NS
	3	252 (112, 572)	<0.0001	225 (123, 568)	<0.0001	NS	240 (61, 582)	<0.0001	268 (69, 523)	<0.0001	NS

Tetanus toxoid (TT) (IU/mL)	1	1.7 (0.7, 3.2)	1.9 (0.8, 3.0)	NS	1.3 (0.6, 3.2)	1.0 (0.4, 2.6)	NS
	3	7.5 (3.2, 10.5)	<0.0001	5.5 (3.8, 15.6)	0.003	NS	6.3 (3.4, 17.8)	<0.0001	6.7 (3.5, 8.1)	<0.0001	NS

*Summary of antibody levels at each visit for polio 1 and 3, hepatitis B, diphtheria toxoid, pertussis toxoid, and TT showing significant boosting of levels in both vaccine groups by visit 3 (+12 weeks). BCG naïve males had higher HepB antibody titers at visit 3 compared to BCG naïve females; this was not the case for the BCG vaccinated males and females. A fitted linear mixed model of log transformed antibody values was used to determine significant differences by vaccine group, bleed and sex. Data shown for *n* = 126 infants (30 BCG vaccinated females, 33 BCG vaccinated males, 28 control females, and 35 control males)*.

## Discussion

The lack of consistent and reliable protection by BCG has galvanized the search for new vaccination strategies using recombinant technology or heterologous vector-based vaccines (42, 43), as a prime-boost to neonatal BCG vaccination. To develop optimal vaccination strategies, it is important to understand the role that the heterologous effects of BCG vaccination play in TB endemic settings. The aim of this study was to analyze *in vitro* reactivity to innate ligands and a panel of common pathogens to see if BCG vaccination of 6-week-old Gambian infants enhanced reactivity to these antigens compared to BCG naïve infants.

One of the surprising findings in this study was a lack of induced *in vitro* reactivity to PPD in the BCG vaccinated infants. However, the BCG vaccinated group had good induction of TST reactivity compared to the naïve group suggesting that the vaccine was effective. In this study, we performed overnight cultures only, whereas we have previously shown that T cell responses to PPD generally peak at 5–7 days of culture (13), thus we probably measured predominantly innate responses to intrinsic PAMPs expressed by mycobacteria in the BCG vaccine (44), which were not boosted by vaccination. Indeed, paradoxically, the BCG vaccinated females had a decline in Th1, Th2, and IL-17 cytokine reactivity to PPD at V3, while the BCG naïve infants experienced an increase in reactivity. This decline may be due to the intervening vaccines given including DTwP that has specifically been shown to suppress innate and adaptive immunity in Gambian females but not males (45). One week is very early for detection of memory T cell responses following BCG vaccination, so this early time point also likely contributed to lack of induced PPD responses. It is also possible that the PPD itself was not immunogenic; however, the same batch was effective in previous studies. We used BCG Russia in this study which has been shown to have poor immunogenicity compared to other strains such as BCG Japan and BCG Denmark (6, 46, 47), which would further contribute to low PPD reactivity in our study. Another explanation is that the mycobacterial response may have been negatively attenuated by environmental bacteria, although our previous studies in infants from the same study area do not support this conclusion (13).

Previous studies have shown that BCG can “train” the innate immune system of healthy European adults *via* epigenetic modification of innate immune cells leading to enhanced pro-inflammatory responses to a range of stimuli (27, 48–51). Furthermore, we have previously found that neonatal BCG vaccination enhanced reactivity to certain TLR ligands in a sex-differential manner, with greater enhancement in females than males (49). However, in this study, we found no evidence of enhanced innate responses (analyzed by TNF-α production) in BCG vaccinated infants in culture supernatants or by ICS. The reason for the discrepancy with previous studies could be due to a number of factors including differences in experimental techniques, the age group at which BCG was given, key genetic differences, and exposure to environmental mycobacteria. Another likely contributory factor is our use of BCG Russia since the previous immune-enhancing studies used BCG Denmark. BCG scar rates predict non-specific beneficial effects, and those immunized with BCG Russia have lower scar rates (approximately 50%) compared to BCG Denmark (72–97%) (6). By implication, this would suggest that BCG Russia is less likely to have beneficial non-specific effects that BCG Denmark (5). This was confirmed in a study conducted in more than 1,000 infants in Uganda in which BCG Denmark was found to have stronger non-specific immunological effects than BCG Russia and BCG Bulgaria (4). Ongoing studies are hoping to confirm this critical issue.

The Th1 polarizing cytokines IL-2, IL-12(p70), and IFN-γ were all affected by vaccine group and sex in this study. The increased CD8^+^ T cell IFN-γ production to *C. albicans* in males and females 1 week after BCG vaccination supports early immune enhancement by the vaccine. There were no other enhanced Th1 type cytokine responses in BCG vaccinated males or females. On the contrary, BCG vaccinated females had decreased IFN-γ:IL-4 to SP 1 week after vaccination.

Many of the significant changes in cytokine reactivity occurred at V3 (+12 weeks) rather than V2 (+1 week). For example, at V3, both BCG vaccinated and naïve infants had decreased Th1 cytokine reactivity and decreased pro- to anti-inflammatory cytokine ratios to certain stimuli. Between V2 and V3 all infants received three doses of OPV, pentavalent vaccine (diphtheria, tetanus, whole cell pertussis, *Haemophilus influenzae* type b, hepatitis B), and PCV-13 at 8, 12, and 16 weeks of age, all of which are likely to have heterologous effects on innate and adaptive immunity. Indeed, we have shown that DTwP vaccination leads to decreased innate and T cell responses in infant females but not males (45), and OPV has also been shown to attenuate immune responses to BCG vaccination (49). Thus, changes in immune reactivity observed in this study may also reflect heterologous effects of these vaccines, and differences between the BCG vaccinated and naïve groups are likely reflect the additional immune modifying effects of BCG. In particular, the negative heterologous effects of DTwP (45) could explain the decline in PPD reactivity for multiple cytokines in BCG vaccinated females but not males at V3.

IL-10 is a key anti-inflammatory cytokine, implicated in the homeostatic regulation of inflammatory responses (41). TLR4-specific IL-10 responses declined in BCG vaccinated females, and IL-10 reactivity to *C. albicans* increased in BCG naïve females at both V2 and V3. BCG naïve males and females also had decreased IFN-γ:IL-10 and TNF-α:IL-10 ratios to a range of stimuli. These results suggest a skewing of immunity toward a more anti-inflammatory profile in those infants who had not received BCG, which was prevented by the prior administration of BCG. Greater anti-inflammatory responses may be detrimental by resulting in less efficient pathogen clearance, and prevention of it by prior administration of BCG could provide an immunological benefit.

Th17 cells have been implicated in protective mycobacterial responses after BCG vaccination (16, 52) and in clearing a range of other pathogens (53). However, we observed high levels of IL-17 production from non-T cells after stimulation with PPD, and thus not a Th17 response; and further found no difference between BCG vaccinated and naïve infants following stimulation with antigens (both vaccine-specific and unrelated) suggesting no effect of BCG vaccination. However, BCG naïve males had evidence of increased IL-17 reactivity to TLR2, while BCG vaccinated females had a decrease, supporting sex-differential enhanced IL-17 immunity in the naïve group.

In summary, we found no evidence that BCG vaccination of 6-week-old Gambian infants alters innate immunity and have shown that Th1 and IL-17 cytokine responses were more likely to increase over time in the BCG naïve individuals. However, the BCG naïve group also had decreased pro- to anti-inflammatory cytokine ratios (IFN-γ:IL-4, IFN-γ:IL-10, and TNF-α:IL-10) and increased IL-10 to certain stimuli, indicating a bias toward an anti-inflammatory response. All of these effects were sex differential, with males and females often showing opposite patterns. This is in keeping with the growing body of literature reporting sex differences in immunity (54). These sex differences are likely caused by a combination of factors including differences in sex hormone levels and expression of X-linked immune response genes. Estrogens are generally immune enhancing and higher in females most of the first year of life; and males undergo a testosterone surge at about 6 weeks of age, which is known to have immunosuppressive effects on immunity (55).

It has been shown that concomitant administration of BCG can enhance antibody responses to other EPI vaccines, including hepatitis B, TT, and OPV (15). Furthermore, certain pneumococcal vaccine serotypes were boosted in infants immunized with BCG at birth indicating that BCG can affect responses to vaccines given later in infancy (35). We did not find a boosting effect of BCG vaccine on the any of the vaccine antibody levels measured. This might be due to the different vaccine strain used in our study as discussed earlier; or the timing of BCG vaccination at 6 weeks of age and thus 6 weeks after their birth HBV and OPV, and 2 weeks before their first pentavalent vaccine (DTP, Hib, and HBV). Importantly, however, protective antibody levels to polio, TT, and hepatitis B were achieved in all subjects regardless of vaccine group, showing that the delay in BCG vaccination was not detrimental to antibody levels in our study. Control males had higher hepatitis B titers than females, which contradicts previous studies showing higher responses among females (54).

Several limitations to this study should be borne in mind. First, the vaccine was administered at 6 weeks of age rather than at birth, yet it is recommended that the vaccine be given at the earliest opportunity after birth. However, in the real life setting, it is estimated that less than half receive BCG in the first month of life, often because BCG comes in multi-dose vials that are not opened until there are sufficient infants to vaccinate (6). Another limitation is the infant numbers assessed since we had limited PBMCs available from study children due to a technical problem with the fresh blood assay. We only cultured overnight which is not optimal for measuring T cell responses since our study was not focused on innate immune stimulation.

In conclusion, BCG Russia given to African infants at 6 weeks of age had no effect on antibody responses to EPI vaccines, or on innate or Th2 cytokine responses to TLR or pathogen stimulation. There was early upregulation of *C. albicans*-specific CD8^+^IFN-γ^+^ responses in BCG vaccinated infants, but a decline in SP reactivity in BCG vaccinated females. By 18 weeks of age, Th1 cytokine responses were downmodulated in BCG vaccinated and naïve infants; TLR-stimulated IL-10 and IL-17 responses declined in BCG vaccinated females; and IL-10 production increased in BCG naïve females. The changes at 18 weeks are likely attributable to immunomodulatory effects of the EPI vaccines given at 8, 12, and 16 weeks of age. However, the more anti-inflammatory immune profile in BCG naïve infants also suggests that prior BCG vaccination may prevent this immune skewing. The results suggest that BCG Russia does not enhance innate immunity in the same way as BCG Denmark, but has a short-lived immune-enhancing effect on CD8^+^ T cell reactivity to *C. albicans* only and possibly deviates the immune system away from an anti-inflammatory IL-10 response. This is the first study to analyze for heterologous effects of BCG Russia, which is important since it is the most widely administered BCG strain worldwide at this time (6).

## Ethics Statement

The Scientific Coordinating Committee (SCC) at the Medical Research Council (MRC) Unit, The Gambia and the MRC-Gambian Government Joint Ethics Committee approved this study (study number SCC 1233). Written informed consent was given by those who agreed to participate in accordance with the Declaration of Helsinki.

## Author Contributions

FD, JS, and KF designed the study; JA oversaw infant recruitment/vaccination/bleeding/clinical assessment; FD and JS did the laboratory assays; FD, JR, MP, MN, SR-J, and KF did the statistical analysis and/or interpreted the data; SH designed the study database and oversaw data entry; all authors critically revised and approved the manuscript and are accountable for the accuracy and integrity of the work.

## Conflict of Interest Statement

The authors declare that the research was conducted in the absence of any commercial or financial relationships that could be construed as a potential conflict of interest.

## Supplementary Material

Figure S1 Gating strategy for CD4 and CD8 T cells by flow cytometry. Lymphocytes were gated on a forward and side scatter plot **(A)**, then singlets **(B)** followed by viable cells by gating out live-dead positive cells **(C)**. Viable lymphocytes were separated into CD3^+^ followed by the CD4^+^ and CD8^+^ populations **(D)**, and the CD3 negative population was identified on a histogram **(E)**.

Figure S2 Gating strategy for intracellular staining by flow cytometry. Representative plots showing the intracellular cytokine production following PMA/ionomycin stimulation for CD3^−^, CD4^+^, and CD8^+^ T cells. Boolean gating was used to quantify cytokine positive cells for the five intracellular cytokines—IL-2, IFN-γ, TNF-α, IL-10, and IL-17.
