# Supplementary material for: Minimal Sex-Differential Modulation of Reactivity to Pathogens and Toll-Like Receptor Ligands following Infant Bacillus Calmette–Guérin Russia Vaccination
Source: Front Immunol. 2017 Sep 8;8:1092. doi: 10.3389/fimmu.2017.01092 (PMC5599783; doi:10.3389/fimmu.2017.01092)
Supplement: Supplementary file 3 [file table_1.docx]

**Supplementary Table 1**

Cytokine levels in supernatants from PBMC cultured in medium (negative control) or PPD. Median values are shown in pg/mL with the interquartile range in brackets.

|  |  |  |  | **IL-2** | **IL-4** | **IL-10** | **IL-12(p70)** | **IL-17** | **IFN-γ** | **TNF-α** |
| --- | --- | --- | --- | --- | --- | --- | --- | --- | --- | --- |
| **Medium** | Baseline | Control | F | 4.7 (0.9-8.4) | 2.4 (0.4-6.4) | 74.4 (9.2-132) | 6.8 (2.7-19.2) | 12.5 (2.3-78.6) | 71.1 (2.1-250) | 252.0 (40.8-547) |
|  |  | Control | M | 4.2 (3.2-11.5) | 2.8 (0.4-9.0) | 21.9 (10.8-75.3) | 11.8 (2.7-32.1) | 40.0 (12.7-140) | 108.1 (40.0-365) | 32.9 (13.4-226) |
|  |  | BCG | F | 4.4 (0.9-10.7) | 2.4 (0.4-6.9) | 16.0 (11.7-56.0) | 6.1 (2.7-26.7) | 27.9 (2.3-112) | 102 (2.1-209) | 54.5 (19.7-305) |
|  |  | BCG | M | 0.9 (0.9-11.1) | 1.1 (0.4-6.2) | 8.6 (1.6-45.6) | 2.7 (2.7-28.2) | 2.3 (2.3-85.3) | 7.0 (2.1-223) | 45.1 (6.7-103) |
|  | + 1 wk | Control | F | 3.7 (1.8-9.5) | 2.8 (0.7-5.4) | 55.0 (15.5-144) | 7.5 (2.7-19.5) | 32.7 (2.3-74.0) | 88.9 (34.3-214) | 253.0 (39.3-648) |
|  |  | Control | M | 4.2 (0.9-12.3) | 2.7 (1.2-8.8) | 33.7 (9.3-89.4) | 13.4 (3.8-39.4) | 50.2 (2.3-114) | 96.8 (21.0-248) | 48.1 (10.4-350) |
|  |  | BCG | F | 4.6 (0.9-10.8) | 3.1 (0.4-9.3) | 27.6 (3.6-78.3) | 15.1 (2.7-34.6) | 59.7 (2.3-107) | 75.4 (5.4-216) | 57.2 (9.5-402) |
|  |  | BCG | M | 4.0 (0.6-9.4) | 2.3 (0.4-7.9) | 32.1 (2.2-61.2) | 5.4 (2.7-32.5) | 37.1 (2.3-106) | 93.1 (3.8-283) | 99.7 (11.5-238) |
|  | + 12 wks | Control | F | 5.8 (3.3-12.3) | 4.0 (0.4-7.8) | 123.5 (52.3-244) | 13.1 (2.7-29.9) | 39.8 (2.3-102) | 167.5 (72.8-254) | 432.1 (205-1053) |
|  |  | Control | M | 7.3 (0.9-15.2) | 1.74 (0.4-8.0) | 54.9 (14.5-115) | 12.8 (2.7-35.8) | 25.3 (3.2-113) | 95.6 (2.1-240) | 161 (40.4-304) |
|  |  | BCG | F | 5.2 (1.0-9.1) | 3.6 (0.4-8.8) | 29.0 (17.5-82.4) | 19.4 (2.7-33.1) | 63.4 (2.3-119) | 93.9 (57.7-237) | 102 (29.9-386) |
|  |  | BCG | M | 5.0 (0.9-7.9) | 2.2 (0.4-8.5) | 45.4 (13.6-117) | 12.3 (2.7-37.5) | 39.6 (2.3-103) | 118 (44.4-259) | 190 (13.5-413) |
| **PPD** | Baseline | Control | F | 6.2 (2.9-11.5) | 2.8 (0.4-6.7) | 69.3 (16.0-183) | 8.2 (2.7-32.1) | 49.7 (2.3-91.8) | 147 (2.1-247) | 260 (23.9-1131) |
|  |  | Control | M | 7.8 (3.4-16.3) | 3.8 (0.5-8.1) | 30.9 (11.4-193) | 11.8 (4.0-32.2) | 61.9 (2.3-134) | 102 (38.2-329) | 121 (12.6-801) |
|  |  | BCG | F | 4.6 (0.9-13.8) | 2.8 (0.4-8.3) | 63.5 (15.7-126) | 6.2 (2.7-24.6) | 54.4 (2.3-115) | 110 (6.2-240) | 365 (19.0-1031) |
|  |  | BCG | M | 4.1 (0.9-8.5) | 0.8 (0.4-6.0) | 13.5 (1.5-85.9) | 4.7 (2.7-28.4) | 2.3(2.3-98.2) | 46.5 (2.1-226) | 30.8 (8.1-55.4) |
|  | + 1 wk | Control | F | 7.5 (2.9-14.7) | 3.8 (1.7-9.3) | 104 (24.7-340) | 12.8 (2.7-33.6) | 64.5 (13.5-106) | 185 (61.1-263) | 383 (132-1895) |
|  |  | Control | M | 8.5 (3.3-18.8) | 6.1 (2.1-10.0) | 54.2 (11.2-227) | 21.0 (9.0-41.3) | 86.7 (40.2-163) | 206 (70.5-432) | 125 (13.2-1095) |
|  |  | BCG | F | 4.4 (0.9-17.9) | 3.5 (0.8-11.0) | 24.9 (10.9-180) | 17.9 (2.7-37.1) | 63.4 (2.3-145) | 92.3 (2.1-357) | 25.1 (13.1-1513) |
|  |  | BCG | M | 7.9 (0.9-14.4) | 4.5 (0.4-8.5) | 42.0 (3.5-176) | 15.4 (2.7-33.1) | 85.7 (2.3-133) | 184 (2.1-351) | 88.5 (10.0-459) |
|  | + 12 wks | Control | F | 5.1 (0.9-9.5) | 2.3 (0.7-7.0) | 235 (111-265) | 13.0 (2.7-28.4) | 20.0 (2.3-101) | 78.1 (2.1-257) | 335 (108-773) |
|  |  | Control | M | 10.4 (2.6-13.0) | 5.6 (0.6-8.9) | 143 (32.0-242) | 12.7 (3.0-38.0) | 73.1 (13.3-118) | 223 (24.8-313) | 580 (123-1399) |
|  |  | BCG | F | 6.5 (1.5-14.7) | 4.2 (0.4-7.3) | 36.4 (20.6-157) | 20.1 (2.7-32.3) | 66.3 (7.5-111) | 94.0 (24.4-172) | 190 (40.7-661) |
|  |  | BCG | M | 6.3 (3.3-10.4) | 6.8 (0.4-9.4) | 69.7 (11.4-220) | 10.8 (2.7-41.3) | 38.3 (2.3-105) | 213 (47.6-293.4) | 872 (15.8-1411) |
