# Supplementary material for: Minimal Sex-Differential Modulation of Reactivity to Pathogens and Toll-Like Receptor Ligands following Infant Bacillus Calmette–Guérin Russia Vaccination
Source: Front Immunol. 2017 Sep 8;8:1092. doi: 10.3389/fimmu.2017.01092 (PMC5599783; doi:10.3389/fimmu.2017.01092)
Supplement: Supplementary file 4 [file table_2.docx]

**Supplementary Table 2**

Cytokine levels in supernatants from PBMC cultured with the TLR ligands HKLM, LPS and CLO-75. Median values are shown in pg/mL with the interquartile range in brackets.

|  |  |  |  | **IL-2** | **IL-4** | **IL-10** | **IL-12(p70)** | **IL-17** | **IFN-γ** | **TNF-α** |
| --- | --- | --- | --- | --- | --- | --- | --- | --- | --- | --- |
| **HKLM** | Baseline | Control | F | 7.9 (5.3-19.6) | 6.4 (2.8-10.5) | 258 (84.2-545) | 22.5 (8.3-37.1) | 72.9 (43.9-152) | 255 (93.1-383) | 753 (380-3097) |
| **(TLR2)** |  | Control | M | 6.8 (3.2-21.4) | 8.7 (0.4-14.7) | 214 (40.7-360) | 25.1 (5.4-44.5) | 84.2 (12.7-205) | 190 (85.6-513) | 860 (47.4-2196) |
|  |  | BCG | F | 7.4 (3.9-16.7) | 6.3 (3.5-10.0) | 157 (22.3-399) | 20.1 (8.6-33.8) | 69.0 (40.8-115) | 121 (80.5-351) | 1021 (21.2-4145) |
|  |  | BCG | M | 4.9 (0.9-11.8) | 2.9 (0.4-5.4) | 78.9 (1.5-262) | 2.7 (2.7-13.4) | 46.8 (10.9-64.1) | 113 (2.1-208) | 444 (12.2-1669) |
|  | + 1 wk | Control | F | 12.3 (6.7-21.6) | 5.9 (3.0-8.8) | 321 (123-657) | 16.6 (9.6-43.6) | 106 (48.9-148) | 259 (192-455) | 2108 (467-3545) |
|  |  | Control | M | 17.3 (5.1-28.8) | 7.6 (5.4-14.9) | 235.8 (37.4-766) | 28.7 (15.9-44.8) | 121 (81.0-202) | 353 (196-588) | 2223 (46.5-4890) |
|  |  | BCG | F | 7.1 (2.8-21.5) | 6.1 (1.0-9.9) | 53.6 (11.4-385) | 16.8 (3.2-45.8) | 74.0 (2.3-153) | 180 (55-474) | 385 (19.8-3213) |
|  |  | BCG | M | 5.7 (0.9-12.2) | 2.6 (0.4-7.5) | 108 (14.4-178) | 5.7 (2.7-15.0) | 35.1 (2.3-66.3) | 91.8 (2.9-272) | 590 (64.5-1695) |
|  | + 12 wks | Control | F | 14.9 (7.8-19.1) | 7.3 (4.6-10.0) | 389 (353-483) | 14.2 (5.6-35.0) | 120 (46.1-133) | 311 (180-380) | 2251 (1141-3030) |
|  |  | Control | M | 16.2 (1.8-20.2) | 8.3 (1.4-12.9) | 268 (70.0-495) | 27.7 (6.5-45.3) | 110 (4.9-209) | 228 (11.1-503) | 1728 (144-2658) |
|  |  | BCG | F | 8.3 (5.9-14.3) | 5.4 (3.5-8.7) | 113 (34.2-332) | 20.1 (2.7-34.6) | 62.0 (27.0-94.5) | 163 (105-225) | 977 (81.5-1796) |
|  |  | BCG | M | 12.6 (4.4-15.3) | 8.3 (2.5-9.2) | 199 (72.5-500) | 10.8 (2.7-52.0) | 64.1 (31.0-159) | 238 (91.2-371) | 1234 (509-2550) |
| **LPS** | Baseline | Control | F | 7.9 (3.9-21.3) | 5.5 (0.9-10.2) | 310 (43.7-471) | 39.3 (14.3-54.6) | 64.8 (2.3-168) | 228 (81.9-418) | 760 (263-3250) |
| **(TLR4)** |  | Control | M | 18.3 (4.3-23.0) | 9.3 (2.4-10.9) | 258 (43.4-380) | 27.7 (14.3-82.0) | 110 (53.2-221) | 304 (98.9-546) | 1585 (19.6-3928) |
|  |  | BCG | F | 8.8 (3.8-28.3) | 7.1 (3.2-13.6) | 172 (22.4-467) | 37.9 (17.4-85.8) | 67.6 (37.1-148) | 183 (93.2-471) | 1548 (62.8-4027) |
|  |  | BCG | M | 4.3 (0.9-13.9) | 2.9 (0.4-8.0) | 25.0 (9.0-334) | 13.8 (2.7-25.6) | 58.4 (2.3-110) | 215 (27.3-319) | 484 (6.7-2587) |
|  | + 1 wk | Control | F | 10.4 (6.5-22.7) | 6.2 (2.8-9.5) | 390 (137-877) | 37.1 (20.9-66.6) | 104 (51.8-155) | 271 (74.0-434) | 1814 (468-3813) |
|  |  | Control | M | 15.8 (6.9-24.7) | 9.8 (5.7-14.7) | 325 (24.8-559) | 41.0 (21.3-67.5) | 138 (80.3-236) | 379 (203-575) | 1949 (29.6-3813) |
|  |  | BCG | F | 8.7 (0.9-25.2) | 5.4 (0.7-11.2) | 56.3 (6.6-464) | 35.3 (3.3-84.4) | 76.6 (2.3-137) | 182 (23.8-391) | 172 (19.9-4626) |
|  |  | BCG | M | 3.3 (0.9-11.6) | 2.4 (0.4-7.8) | 30.1 (1.5-265.2) | 13.7 (2.7-43.5) | 51.2 (2.3-131) | 83.6 (2.1-340) | 153 (15.5-1433) |
|  | + 12 wks | Control | F | 10.8 (6.7-18.8) | 6.2 (3.9-8.4) | 415 (301-529) | 18.7 (8.0-44.6) | 75.9 (16.8-106) | 240 (140-302) | 1686 (377-3004) |
|  |  | Control | M | 8.2 (1.7-18.6) | 5.1 (0.9-12.6) | 236 (68.0-540) | 40.2 (5.9-67.9) | 71 (8.7-172) | 178 (11.1-379) | 787 (92.3-1804) |
|  |  | BCG | F | 6.5 (3.5-19.0) | 7.1 (3.1-10.2) | 110 (32.6-310) | 26.4 (18.5-48.5) | 69.0 (45.5-131) | 143 (85.2-335) | 822 (63.1-1765) |
|  |  | BCG | M | 9.1 (3.9-14.4) | 5.9 (1.3-8.5) | 248 (44.4-629) | 25.8 (11.6-45.1) | 56.1 (2.3-134) | 201 (60.0-354) | 968 (498-1421) |
| **CLO-75** | Baseline | Control | F | 23.3 (8.2-34.7) | 9.3 (4.9-13.9) | 237 (44.4-450) | 34.7 (14.9-51.8) | 121 (84.2-205) | 422 (230-509) | 4361 (1309-6738) |
| **(TLR7/8)** |  | Control | M | 12.1 (3.9-40.2) | 8.5 (1.2-19.6) | 140 (42.7-407) | 34.2 (6.1-84.7) | 106 (57.8-317) | 276 (118-881) | 2659 (63.0-6112) |
|  |  | BCG | F | 14.3 (4.9-37.8) | 11.3 (3.9-16.3) | 144 (20.5-411) | 36.5 (17.8-71.3) | 115 (67.9-159) | 340 (98.1-729) | 2245 (91.5-8624) |
|  |  | BCG | M | 4.9 (0.9-25.0) | 2.8 (0.4-10.9) | 27.2 (9.0-199) | 16.0 (2.7-24.4) | 58.4 (2.3-178) | 252 (2.1-513) | 587 (15.1-4922) |
|  | + 1 wk | Control | F | 25.6 (6.8-39.4) | 9.5 (3.1-14.6) | 320 (71.3-578) | 46.7 (15.6-80.6) | 157 (53.3-239) | 434 (212-682) | 4331 (1277-8457) |
|  |  | Control | M | 24.0 (5.0-35.0) | 9.3 (4.8-18.3) | 209 (23.8-643) | 38.3 (21.6-98.7) | 142 (76.4-315) | 462 (173-848) | 3021 (35.6-7615) |
|  |  | BCG | F | 12.4 (1.8-30.1) | 6.6 (2.1-15.8) | 47.0 (11.4-373) | 31.2 (4.4-67.1) | 91.4 (10.0-198) | 293 (65.7-454) | 1022 (27.3-6075) |
|  |  | BCG | M | 5.7 (0.9-25.8) | 2.8 (0.4-12.0) | 27.0 (1.9-252) | 14.0 (2.7-37.1) | 57.2 (2.3-214) | 75.2 (2.1-577) | 708 (83.4-3397) |
|  | + 12 wks | Control | F | 18.7 (6.8-29.2) | 7.8 (5.7-12.9) | 310 (170-484) | 42.3 (21.1-53.6) | 111 (88.6-193.8) | 306 (211-621) | 3557 (806-5421) |
|  |  | Control | M | 22.2 (9.1-28.2) | 11.3 (4.5-16.9) | 164 (50.2-408) | 51.6 (25.2-81.0) | 151 (81.3-244) | 472 (123-801) | 3420 (1151-4189) |
|  |  | BCG | F | 10.0 (4.7-16.0) | 7.1 (4.9-9.7) | 111 (25.4-301) | 32.5 (19.2-51.2) | 87.1 (54.3-138) | 240 (127-496) | 2135 (155-4544) |
|  |  | BCG | M | 15.0 (6.8-27.7) | 7.5 (2.3-13.4) | 184 (54.0-368) | 39.3 (19.4-59.1) | 112 (44.3-208) | 319 (131-690) | 2084 (938-3946) |
