# Supplementary material for: Minimal Sex-Differential Modulation of Reactivity to Pathogens and Toll-Like Receptor Ligands following Infant Bacillus Calmette–Guérin Russia Vaccination
Source: Front Immunol. 2017 Sep 8;8:1092. doi: 10.3389/fimmu.2017.01092 (PMC5599783; doi:10.3389/fimmu.2017.01092)
Supplement: Supplementary file 5 [file table_3.docx]

**Supplementary Table 3**

Cytokine levels in supernatants from PBMC cultured with the heat killed pathogens *C. albicans* (CA), *E. coli* (EC) and *S. pneumoniae* (SP). Median values are shown in pg/mL with the interquartile range in brackets.

|  |  |  |  | **IL-2** | **IL-4** | **IL-10** | **IL-12(p70)** | **IL-17** | **IFN-γ** | **TNF-α** |
| --- | --- | --- | --- | --- | --- | --- | --- | --- | --- | --- |
| **CA** | Baseline | Control | F | 4.7 (1.5-7.2) | 2.6 (0.4-5.6) | 50.7 (18.6-70.0) | 10.8 (2.7-30.5) | 48.5 (2.3-95.4) | 69.4 (18.6-224.1) | 81.9 (50.0-323) |
|  |  | Control | M | 4.0 (1.3-11.0) | 2.6 (0.4-8.7) | 27.6 (13.4-90.6) | 12.4 (3.0-32.4) | 47.9 (2.3-117) | 136 (11.1-270) | 138 (29.8-406) |
|  |  | BCG | F | 3.6 (0.9-7.2) | 2.5 (0.4-5.4) | 26.2 (17.5-61.2) | 9.7 (2.7-25.0) | 29.2 (2.3-75.2) | 105 (2.1-188) | 98.6 (15.5-559) |
|  |  | BCG | M | 3.8 (0.9-10.6) | 2.3 (0.4-8.4) | 18.5 (1.5-45.2) | 12.4 (2.7-32.7) | 23.0 (2.3-117) | 49.1 (20.8-342) | 48.1 (13.6-162) |
|  | + 1 wk | Control | F | 4.3 (0.9-9.4) | 2.7 (0.4-7.8) | 55.7 (26.0-189) | 8.3 (2.7-32.1) | 36.9 (2.3-82.8) | 90.9 (12.7-237) | 154 (48.0-654) |
|  |  | Control | M | 7.3 (3.6-12.8) | 3.3 (2.4-9.2) | 37.2 (13.6-151) | 17.0 (7.9-36.9) | 60.1 (24.9-129) | 165 (71.6-347) | 125 (13.3-576) |
|  |  | BCG | F | 4.2 (0.9-7.1) | 3.3 (0.4-8.9) | 20.4 (8.0-75.2) | 19.2 (2.7-41.0) | 53.0 (2.3-110) | 61.7 (2.1-238) | 23 (7.7-266) |
|  |  | BCG | M | 5.5 (0.9-15.9) | 2.7 (0.4-7.5) | 31.1 (4.1-119) | 13.2 (2.7-36.4) | 37.9 (2.3-130) | 144 (4.5-349) | 121 (8.6-393) |
|  | + 12 wks | Control | F | 6.0 (1.2-13.5) | 2.5 (0.4-9.3) | 244 (62.0-312) | 9.2 (2.7-35.0) | 22.7 (2.3-101) | 90.0 (42.2-259) | 436 (213-995) |
|  |  | Control | M | 5.4 (2.1-9.8) | 3.7 (0.4-9.1) | 65.3 (15.7-183) | 25.7 (3.7-43.6) | 36.9 (3.2-119) | 95.4 (20.3-256) | 95.5 (34.9-543) |
|  |  | BCG | F | 3.0 (0.9-6.5) | 4.0 (0.4-7.1) | 23.7 (12.4-51.9) | 11.5 (2.7-24.9) | 16.7 (2.3-55.5) | 78.7 (34.8-134) | 146 (44.6-215) |
|  |  | BCG | M | 4.7 (0.9-7.5) | 2.8 (0.4-8.8) | 56.2 (30.4-131) | 20.7 (4.4-33.7) | 38.3 (2.3-104) | 156 (13.0-253) | 170 (85.2-467) |
|  | Baseline | Control | F | 12.9 (2.0-22.7) | 3.9 (2.2-10.0) | 307 (18.4-413) | 28.4 (2.8-50.1) | 80.6 (2.3-161) | 304 (20.2-445) | 1564 (51.3-3559) |
| **EC** |  | Control | M | 6.4 (3.7-20.8) | 4.0 (1.3-12.2) | 132 (18.1-356) | 16.8 (8.7-57.2) | 86.2 (19.5-128) | 215 (28.8-483) | 354 (37.1-2573) |
|  |  | BCG | F | 8.0 (4.6-17.5) | 8.4 (2.5-11.0) | 103 (12.4-217) | 33.1 (14.0-40.1) | 79.9 (17.3-115) | 159 (66.2-344) | 611 (17.4-1847) |
|  |  | BCG | M | 8.0 (0.9-14.4) | 5.6 (0.4-9.3) | 37.6 (2.0-235) | 14.8 (4.9-37.2) | 88.7 (2.3-154) | 147 (2.1-481) | 65.6 (6.7-1847) |
|  | + 1 wk | Control | F | 15.9 (6.8-23.6) | 6.7 (3.9-10.6) | 340 (164-565) | 37 (17.8-71.6) | 114 (2.3-136) | 320 (108-424) | 1900 (1049-4469) |
|  |  | Control | M | 11.5 (3.5-21.2) | 6.4 (2.2-9.5) | 145 (13.3-430) | 32.2 (14.7-66.0) | 95.4 (46.2-152) | 217 (114-473) | 667 (13.9-2986) |
|  |  | BCG | F | 6.7 (1.6-17.0) | 4.7 (0.4-9.9) | 38.0 (7.8-280) | 28.9 (4.9-54.6) | 67.2 (12.4-146) | 148 (15.5-343) | 76.2 (9.1-4306) |
|  |  | BCG | M | 9.4 (0.9-22.3) | 7.5 (0.4-11.4) | 55.4 (7.1-303) | 25.7 (3.4-46.2) | 101 (2.3-154) | 269 (4.6-508) | 599 (12.0-2902) |
|  | + 12 wks | Control | F | 6.3 (5.4-18.6) | 5.7 (1.5-8.7) | 504 (312-602) | 32.1 (19.3-48.1) | 92.9 (45.4-114) | 226 (126-335) | 1036 (98.1-2795) |
|  |  | Control | M | 11.7 (5.0-22.0) | 7.2 (0.4-15.6) | 229 (27.8-581) | 38.8 (4.7-85.1) | 73.0 (2.3-206) | 272 (29.2-462) | 2145 (292-2976) |
|  |  | BCG | F | 5.8 (2.4-8.7) | 4.8 (0.8-6.2) | 67.2 (28.6-188) | 23.4 (13.8-41.5) | 54.0 (2.3-92.4) | 171 (32-222) | 562 (123-1583) |
|  |  | BCG | M | 10.1 (4.5-12.3) | 6.4 (1.0-8.8) | 173 (55.6-478) | 33.0 (10.8-50.8) | 81.5 (38.8-125) | 276 (161-341) | 1277 (248-2279) |
| **SP** | Baseline | Control | F | 7.7 (3.7-10.2) | 4.8 (2.0-6.5) | 149 (27.0-185) | 13.5 (2.7-37.2) | 53.2 (30.1-114) | 184 (52.0-286) | 774 (148-1199) |
|  |  | Control | M | 6.8 (2.5-17.9) | 6.3 (1.5-10.0) | 39.7 (14.3-205) | 20.0 (9.9-42.6) | 69.2 (27.8-146) | 173 (36.7-435) | 88.4 (19.6-1624) |
|  |  | BCG | F | 4.1 (2.7-7.2) | 3.8 (0.8-9.9) | 42.2 (15.4-141) | 13.8 (4.1-27.2) | 66.2 (2.3-110) | 107 (2.3-110) | 145 (18.3-855) |
|  |  | BCG | M | 4.9 (0.9-14.3) | 2.8 (0.4-8.7) | 21.3 (1.5-143) | 8.6 (2.7-32.9) | 37.3 (2.3-122) | 101 (2.3-351) | 44.3 (7.3-964) |
|  | + 1 wk | Control | F | 7.3 (3.5-9.7) | 3.9 (2.1-7.3) | 259 (43.2-395) | 8.3 (2.7-26.7) | 56.3 (2.3-101) | 164 (57.2-248) | 888 (151-1724) |
|  |  | Control | M | 8.0 (3.5-16.8) | 5.7 (2.4-11.4) | 64.9 (12.1-284) | 23.5 (13.3-36.6) | 92.2 (58.7-138) | 212 (94.5-398) | 100 (12.9-1847) |
|  |  | BCG | F | 4.2 (0.9-10.4) | 3.1 (0.4-9.8) | 29.3 (10.5-140) | 20.8 (2.7-36.7) | 69.7 (2.3-115) | 94.0 (2.1-262) | 22.9 (8.4-977) |
|  |  | BCG | M | 5.0 (0.9-14.9) | 4.4 (0.4-8.4) | 40.7 (3.0-264) | 15.4 (2.7-38.4) | 90.0 (2.3-133) | 218 (2.1-372) | 83.6 (9.8-1086) |
|  | + 12 wks | Control | F | 6.7 (0.9-10.6) | 4.8 (0.9-9.2) | 330 (101-426) | 19.2 (2.7-36.0) | 50.4 (2.3-105) | 188 (23.8-242) | 262 (46.1-1151) |
|  |  | Control | M | 6.8 (0.9-18.0) | 5.8 (0.6-10.5) | 131 (16.2-420) | 24.5 (4.7-39.2) | 87.1 (2.3-146) | 171 (2.1-370) | 518 (37.9-1637) |
|  |  | BCG | F | 2.8 (0.9-5.9) | 2.2 (0.4-7.7) | 27.0 (16.9-64.6) | 12.1 (2.7-30.5) | 29.2 (2.3-84.3) | 84.3 (34.6-159) | 176 (28.9-319) |
|  |  | BCG | M | 4.9 (1.2-11.2) | 7.3 (0.4-9.3) | 173 (45.8-289) | 27.5 (4.0-40.1) | 54.3 (2.3-126) | 166 (24.6-267) | 559 (139-989) |
